# Determination of pH Effects on Phosphatidyl-Hydroxytyrosol and Phosphatidyl-Tyrosol Bilayer Behavior

**DOI:** 10.3390/mps1040041

**Published:** 2018-11-09

**Authors:** Kervin O. Evans, David L. Compton, Michael Appell

**Affiliations:** 1 Renewable Product Technology Research Unit, United States Department of Agriculture Research Service, National Center for Agricultural Utilization Research, 1815 N. University Street, Peoria, IL 61604, USA; david.compton@ars.usda.gov; 2 Mycotoxin Prevention and Applied Microbiology, United States Department of Agriculture Research Service, National Center for Agricultural Utilization Research, 1815 N. University Street, Peoria, IL 61604, USA; michael.appell@ars.usda.gov

**Keywords:** method application, bilayer, phospholipid, dynamic light scattering, zeta potential

## Abstract

A robust method was developed to investigate the liposomal behavior of novel enzymatically-synthesized hydroxytyrosol and tyrosol phospholipids. Bilayer characteristic obtained by this method, including bilayer formation stability and adsorption properties, were explored using dynamic light scattering, zeta-potential measurements, and quartz crystal microbalance with dissipation monitoring (QCMD), respectively. Liposome diameters were found to typically increase from pH 5.5 to pH 10. Zeta potentials values, on the other hand, were found to be well below −25 mV at all pH conditions explored, with the lowest values (and thus, the best liposome stability) at pH 5.5 or pH 10. Quartz crystal microbalance with dissipation monitoring measurements demonstrated that 100% 1,2-dioloeoylphosphatidyl-hydroxytyrosol (DOPHT) liposomes adsorbed intact onto silica in buffer conditions at pH 5.5 and with no calcium, or at pH 7.5 with calcium (no adsorption was detected at pH 10). 1,2-Dioleoylphosphatidyl-tyrosol (DOPT) liposomes were shown to adsorb intact under buffer conditions only at pH 5.5 with and without calcium. 1,2-Dioleoylphosphatidyl-2-phenolethanol (DOPPE), in comparison, readily adsorbed intact at pH 7.5 without calcium and just slightly at pH 5.5 with calcium present, but formed a supported bilayer over hours at pH 5.5 in the absence of calcium ions.

## 1. Introduction

Hydroxytyrosol and tyrosol are phenolic compounds found abundantly in olive oil. Their presence in the traditional Mediterranean diet suggests that hydroxytyrosol and tyrosol may have health benefits [1]. These health benefits may arise from their antioxidant properties, making them highly desirable for applications within the food and health industries [2,3]. It is thus vital for both hydroxytyrosol and tyrosol to have the ability to partition or mix well with different media.

Previous studies have demonstrated the ability to esterify tyrosol and hydroxytyrosol either chemically [4,5] or enzymatically [6], such that the tyrosol-based esters each were more lipophilic and maintained antioxidant properties. Further research has shown that tyrosol and hydroxytyrosol esters enzymatically synthesized from cuphea oil readily mixed with phospholipids to form liposomes [6,7]. Our previous research further demonstrated that hydroxytyrosol and tyrosol could be enzymatically transphosphatidylated into tyrosol-based phospholipids that readily formed liposomes at pH 7.5 [8]. It was further shown that tyrosol-based liposomes were typical 85 nm in diameter and maintained a zeta potential less than −25 mV at pH 7.5, minus any salts. Moreover, the previous study was able to demonstrate that, typically, liposomes containing fractions of tyrosol-based phospholipids formed a supported liposome layer and/or a supported bilayer on silica in a buffer containing 100 mM NaCl and usually 2 mM CaCl_2_. The current work further explored bilayer properties of tyrosol-based phospholipid liposomes under high and low pH (pH 10 vs. pH 5.5) buffer conditions, instead just at pH 7.5. Specifically, this work explored the size, zeta potential and silica adhesion properties under salt conditions (100 mM NaCl, ±2 mM CaCl_2_) of liposomes composed of 100% tyrosol-based phospholipids at pH 10 and pH 5.5. This will allow for determining the influence of pH on the stability and bilayer forming capability of liposomes composed solely of tyrosol-based phospholipids. Phospholipids with hydroxytyrosol (two hydroxyl groups on a phenyl ring), tyrosol (phenyl ring with one hydroxyl group), and 2-phenyl ethanol (DOPPE—phenyl ring with no hydroxyl group) headgroups were compared to ascertain whether the hydroxyl group was key to liposome adsorption.

## 2. Materials and Methods

### 2.1. Materials

The following agents were purchased accordingly: 1,2-Dioleoyl-*sn*-glycero phosphocholine (DOPC) and 1,2-dioleoyl-*sn*-glycero-3-[phospho-*rac*-(1-glycerol)] (DOPG) from Avanti Polar Lipids, Inc. (Alabaster, AL, USA); *Streptomyces* sp. phospolipase D (PLD) was from Enzo Life Sciences (Farmingdale, NY, USA); hydroxytyrosol or 2-(3,4-dihydroxyphenyl)ethanol from TCI American (Portland, OR, USA); tyrosol or 2-(4-hydroxyphenyl)ethanol from Sigma-Aldrich (St. Louis, MO, USA); American Chemical Society (ACS) grade buffers and sodium chloride from Fisher Scientific (Hampton, NH, USA).

### 2.2. 1,2-Dioleoylphosphatidyl-tyrosol (DOPT) and 1,2-Dioleoylphosphatidyl-hydroxytyrosol (DOPHT) Lipid Synthesis, Isolation and Verification

Phosphatidyl-tyrosol and phosphatidyl-hydroxytyrosol synthesis, isolation, and verification procedures were done as previously published [8]. First, tyrosol and DOPC was combined at a 8.5:1 *w*/*w* ratio in ethyl acetate and a 80 mM CaCl_2_, 200 mM sodium acetate buffer at pH 5.6. Phospholipase D (PLD) was then added to this mix and stirred at 32 °C under argon for 20 h. The mixture was phase separated to remove the ethyl acetate, followed by drying under argon. The resulting dried material was sonicated for 1 min in chloroform and filtered prior to flash chromatography analysis. The flash chromatography was done with a CombiFlash Rf200i system (Teledyne Isco, Lincoln, NE, USA) set up a 100% ethyl acetate-to-100% methanol gradient on a silica column (4 g RediSepRf Gold, Teledyne Isco). Pure material was verified using silica gel plates exposed to 7/30/35/35 *v*/*v* water/chloroform/trielthylamine/ethanol for thin-layer chromatography and nuclear magnetic resonance (NMR) [8].

### 2.3. Liposome Preparation

Liposomes were created using the dried film method [9]. The DOPHT stored in chloroform was aliquoted into 4½-mL amber vials and dried to a film under a stream of argon and continually dried under vacuum overnight; DOPT stored in a chloroform/methanol 2:1 mixture was also dried to a film under argon and further dried under an overnight vacuum. Overnight vacuum was accomplished using a speed-vacuum condenser equipped with a Savant SPD131DDA SpeedVac concentrator and a RVT4104 refrigerated vapor trap (Thermo Fisher, Rockford, IL, USA). Dried lipid films were then hydrated in a buffer at the appropriate pH and periodically vortexed for nearly an hour. Hydrated lipids were then subjected to five cycles of freezing (ethanol on dry ice) and thawing (50 °C water bath) just prior to extrusion through double-stacked 100 nm pore filter paper housed in a LiposoFast hand-held extruder (Avestin, Inc.; Ottawa, ON, Canada). Liposomes were passed 11 times back and forth through the filters.

### 2.4. Dynamic Light Scattering and Zeta Potential

Size and surface charge of liposomes were characterized at 25 °C and the appropriate pH using a Zetasizer nano ZS system (Malvern Instruments, Malvern, UK). The Zetasizer is equipped with a 633 nm laser and optical detection was done at a 90° scattering angle. The buffers were either 10 mM MES, pH 5.5; 10 mM HEPES, pH 7.5; or 10 mM CHES, pH 10. Each measurement consisted of three cycles of 15 scans lasting 20 s for sizing data collection and three iterations of 100 scans lasting 20 s for zeta potential data. All measurements were conducted in triplicate and the average was reported. All data was collected and processed using the Zetasizer nano software provided by Malvern Instruments. 

### 2.5. Quartz Crystal Microbalance with Dissipation Monitoring (QCMD)

Liposome adsorption behavior on silica was monitored at 23 °C using a QCMD unit (Biolin Scientific, Västra Frölunda, Sweden). The QCMD uses the piezoelectric properties of AT-cut (quartz cut at an angle of 35°25′ to the z-axis) quartz crystals; under the influence of an alternating current these crystals vibrate at a resonance frequency (typically ~5 MHz). Material adsorbing onto the crystal causes the vibration of the crystal to shift to a lower frequency called a negative frequency shift. The more material adsorbs onto the crystal, the greater the mass of that material becomes on the crystal. The greater the mass, the greater the frequency shift. Assuming that each location on the crystal has the same probability for material to adsorb, that the adsorbed materials is a thin layer and it does not slip then the mass of the adsorbed material is proportional to the frequency change and is described by Sauerbrey equation: ∆m = −C × ΔF/n where n is the overtone number (1st, 2nd, 3rd, …, 13th), Δf is the frequency shift (Hz) and C is the mass-sensitivity constant (17.7 ng/cm^2^/Hz). Simultaneously, frequency shifts and dissipation shifts were measured for each overtone.

Crystals were placed in a holder and soaked in 2% (*v*/*v*) sodium dodecyl sulfate aqueous solution for cleaning. After 30 min, the crystals were thoroughly rinsed with nanopore water and dried using pure nitrogen. Dried crystals were then placed into an ultraviolet (UV)/Ozone cleaner for 10 min to remove any residual contaminants and put through a final rinsing and drying. Buffers were drawn across crystals using a peristalic pump as the signal stabilized to less than 1 Hz shifts. Crystals submerged in the appropriate buffer were exposed to low concentration (~50 µM) liposomes solutions at a flow rate of 75 µL/min. Crystals were rinsed with buffer to remove excess liposomes after adsorption and/or rupture appeared complete. The buffers contained either 10 mM 2-(*N*-morpholino)ethanesulfonic acid (MES), 100 mM NaCl or 2 mM CaCl_2_, 10 mM MES, 100 mM NaCl for pH 5.5; 10 mM 2-[4-(2-hydroxyethyl)piperazin-1-yl]ethanesulfonic acid (HEPES), 100 mM NaCl or 2 mM CaCl_2_, 10 mM HEPES, and 100 mM NaCl for pH 7.5; or 10 mM 2-cyclohexylamino)ethanesulfonic acid (CHES), 100 mM NaCl or 2 mM CaCl_2_, 10 mM CHES, and 100 mM NaCl for pH 10. The calcium chloride concentration chosen was based on the concentration demonstrated to induce and/or speed up supported bilayer formation by Richter, et al., 2006 [10], especially for liposomes with negative surface potentials. Frequency and dissipation changes were monitored for the 3rd, 5th, 7th, 9th, 11th, and 13th overtones. 

## 3. Results and Discussion

### 3.1. Liposome Size and Zeta Potential Characterization

**Figure 1:**
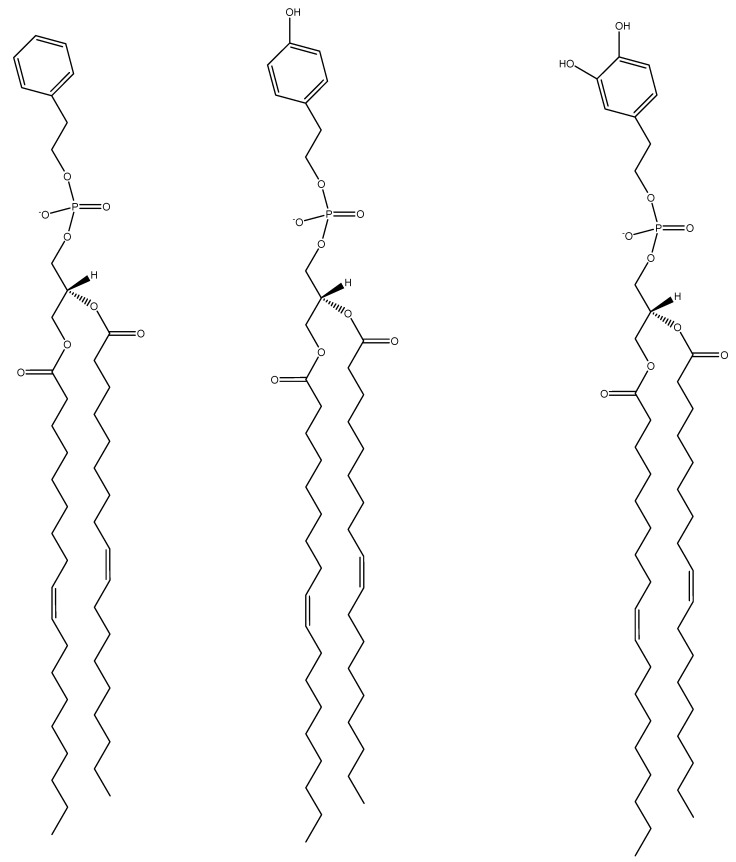
Structural representation of 1,2-dioleoylphosphatidyl-2-phenolethanol (DOPPE), 1,2-dioleoylphosphatidyl-tyrosol (DOPT) and 1,2-dioloeoylphosphatidyl-hydroxytyrosol (DOPHT) (left to right) phospholipids.

**Figure 2:**
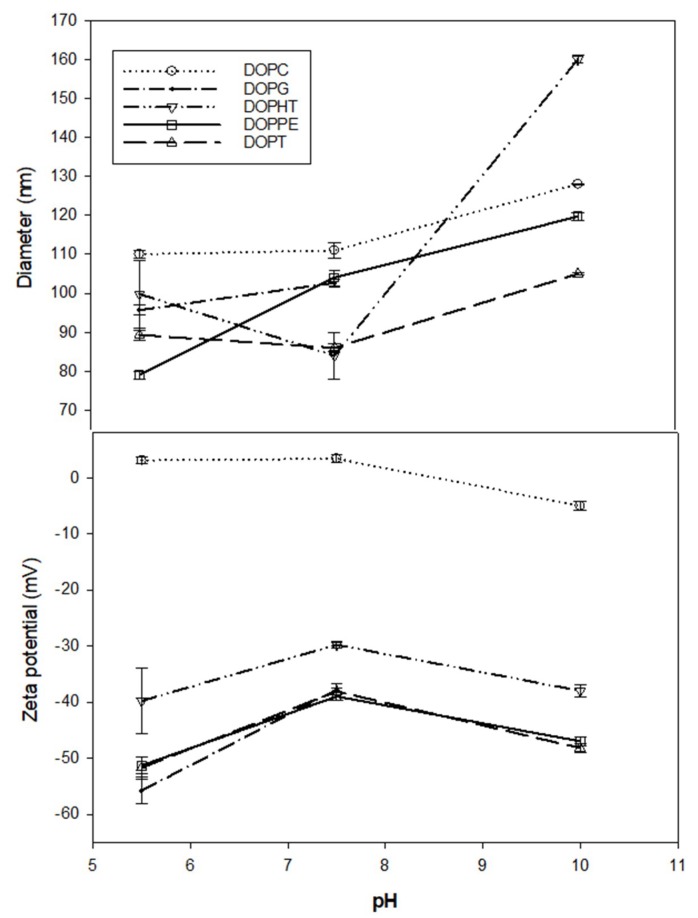
Liposome size (**top panel**) and zeta potential (**bottom panel**) as a function of buffer pH. 1,2-dioleoyl-*sn*-glycero phosphocholine (DOPC); 1,2-dioleoyl-*sn*-glycero-3-[phospho-*rac*-(1-glycerol)] (DOPG).

Liposomes formed from 100% DOPHT, DOPT, or DOPPE (Figure 1) were compared under buffer conditions of pH 5.5, 7.5 and 10. These liposomes were also compared to those liposomes formed from zwitterionic (DOPC) or negatively charged (DOPG) lipids under the same buffer conditions. The buffers excluded NaCl to eliminate any influences due to salt. First, all newly synthesized phospholipids (DOPHT, DOPPE and DOPT) liposomes exhibited increased diameters under pH 10 conditions as compared to their diameters at pH 5.5 or 7.5 (Figure 2, top panel). The fact that each set of liposomes had increasing diameters over the pH range of 5.5 to 10 fits well with Petelska and Figaszewski’s 2002 findings that increasing pH reduces membrane tension [11], because larger liposomes have lower curvature stress than smaller liposomes, which translates into reduced membrane tension [12].

Most notable was that DOPHT liposomes were smaller at pH 7.5 than at pH 5.5, but were nearly double in diameter at pH 10 compared to pH 7.5. The DOPPE liposomes, on the other hand, demonstrated a continued increase in diameter over the entire pH range and DOPT liposomes relatively maintained size (~90 nm) at pH 5.5 and pH 7.5 before forming larger liposomes (~100 nm) at pH 10.

The zeta potential characteristics of DOPHT, DOPPE, and DOPT liposomes, in contrast, peaked at pH 7.5, with the highest (least negative) value and the lowest values at both pH 5.5 and pH 10 (Figure 2, bottom panel). This indicates that DOPHT, DOPPE, and DOPT liposomes are most stable at pH 5.5 and pH 10, but maintained high stability at all pH values explored. This was consistent with highly-stable nanoparticles described as having zeta potentials either ≥25 mV or ≤−25 mV [12]. The DOPC liposomes, for comparison, proved to maintain virtually the same surface charge and size at low and medium pH (pH 5.5 and pH 7.5, respectively), whereas they demonstrated a reduced surface charge (~−5 mV) and an increased size (~128 nm) at high pH (pH 10). Liposomes made from DOPG, on the other hand, exhibited an increase in size over the pH range, and an increase in surface charge that was well below −25 mV at all pH values investigated.

### 3.2. The DOPHT, DOPPE and DOPT Bilayer Behavior at Various pH Levels

**Figure 3:**
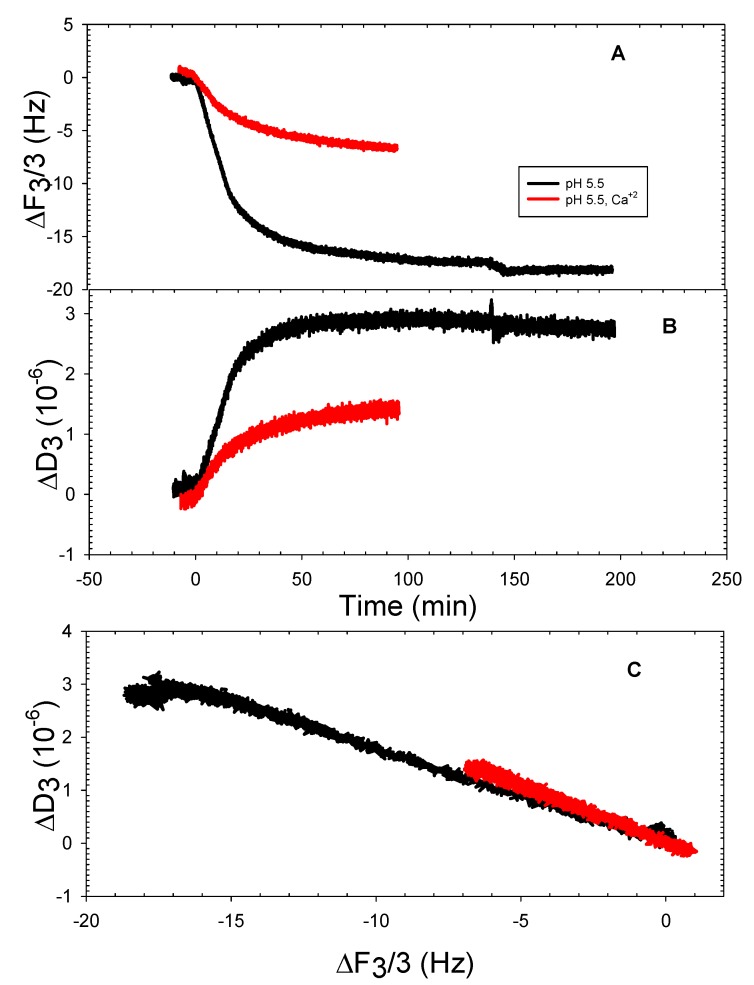
Representative quartz crystal microbalance with dissipation (QCMD) frequency (**A**) and dissipation (**B**) shifts of DOPHT adsorption onto silica at pH 5.5 in the absence and presence of 2 mM CaCl_2_; ΔF vs. ΔD plot (**C**).

It has been demonstrated quite effectively that quartz crystal microbalance with dissipation monitoring (QCMD) is an excellent tool for characterizing typical properties of liposomes adsorbed onto silica surfaces [9,13,14,15]. Previously, it was demonstrated that when buffer conditions were at pH 7.5 with 100 mM NaCl present, 100% DOPHT liposomes only adsorbed slightly in the presence of calcium ions, and 100% DOPT liposomes adsorbed slightly in the absence of calcium ions and supported bilayers formed from both only when there was no more than 25 mole percent of either present within the lipid matrix and calcium ions in the buffer [8]. The purpose of the QCMD measurements here was to ascertain whether 100% DOPHT, DOPT, and DOPPE liposomes adsorbed and/or formed supported bilayers on silica at pH 5.5 or pH 10, and whether 2 mM CaCl_2_ improved the process. Estimated pKa values for DOPT and DOPHT showed that DOPT has a value of nearly 9.4 and DOPHT was approximately 10.2, indicating that full deprotonation does not occur unless buffers are near pH 9 or 10, respectively. This being the case, DOPHT and DOPT liposomes were monitored for adsorption behavior at buffer pH 5.5 and 10. Figure 3 shows the adsorption behavior of DOPHT at pH 5.5 (no adsorption was detected at pH 10). Adsorption of DOPHT liposomes at pH 5.5 occurred both in the absence and in the presence of calcium ions (Figure 3A). It was shown that the frequency shift for liposomes without calcium present was nearly three times greater than that for liposomes in the presence of calcium. Comparing the slope of frequency-change vs. dissipation change (∆F-∆D) plot for DOPHT at pH 7.5 presented in [8] and for DOPHT at pH 5.5 presented here shows that the slope for DOPHT adsorption at pH 7.5 occurred at a faster rate than either conditions for DOPHT at pH 5.5. This is consistent with properties of the liposomes formed at pH 7.5 being smaller than those formed at pH 5.5, as smaller DOPHT liposomes are expected to be under greater interfacial tension, and have been shown to adsorb faster to silica than larger liposomes [16]. This also suggests that pH 7.5 is nearer to the optimal pH for interfacial tension for DOPHT according to the data of Petelska and Figaszewski, 2002 [11].

Given that liposomes in both instances were virtually the same sizes, the frequency shift differences suggest that more DOPHT liposomes were deposited and adsorbed in the absence of calcium ions than in the presence of calcium at pH 5.5. This is also borne out in Figure 3B, where final dissipation shift values were nearly three times greater for DOPHT liposomes in the absence of calcium ions than liposomes in the presence of calcium ions. Figure 3C shows that despite there being a difference in the amount of liposomes absorbed onto silica in the presence versus the absence of calcium ions at pH 5.5, the process of adsorption was the same, and there was no structural deformation or rearrangement of liposomes; this was indicative of the linearity of the ΔF-ΔD plots [17].

**Figure 4:**
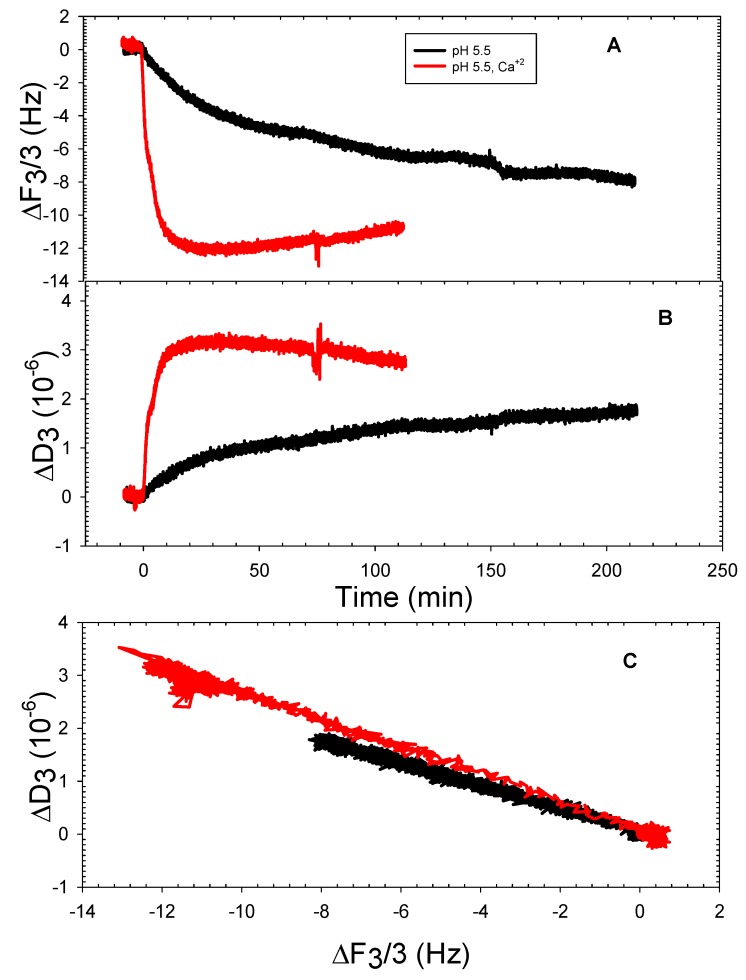
Representative QCMD frequency (**A**) and dissipation (**B**) shifts of DOPT adsorption onto silica at pH 5.5 in the absence and presence of 2 mM CaCl_2_; ΔF vs. ΔD plot (**C**).

The DOPT liposomes adsorption behavior at pH 5.5 was opposite to that of DOPHT liposomes at pH 5.5, as illustrated in Figure 4. The presence of calcium ions caused nearly 1.5 times the amount of DOPT liposomes to deposit onto silica than the amount deposited in the absence of calcium ions, as shown by frequency and dissipation shifts (Figure 4A,B). The ΔF-ΔD plots (Figure 4C) of DOPT liposomes deposition onto silica, however, indicated that both conditions resulted in liposomes also depositing at similar rates without deformation or rearrangement, as shown for DOPHT liposomes. There was no deposition detected for DOPT liposomes at pH 10. The comparison of the slopes of ΔF-ΔD plots of DOPT at pH 7.5 [8] also reveals that DOPT liposomes at pH 7.5 adsorbed at a faster rate than those at pH 5.5, indicating too that interfacial tension is greatest for DOPT at pH 7.5.

**Figure 5:**
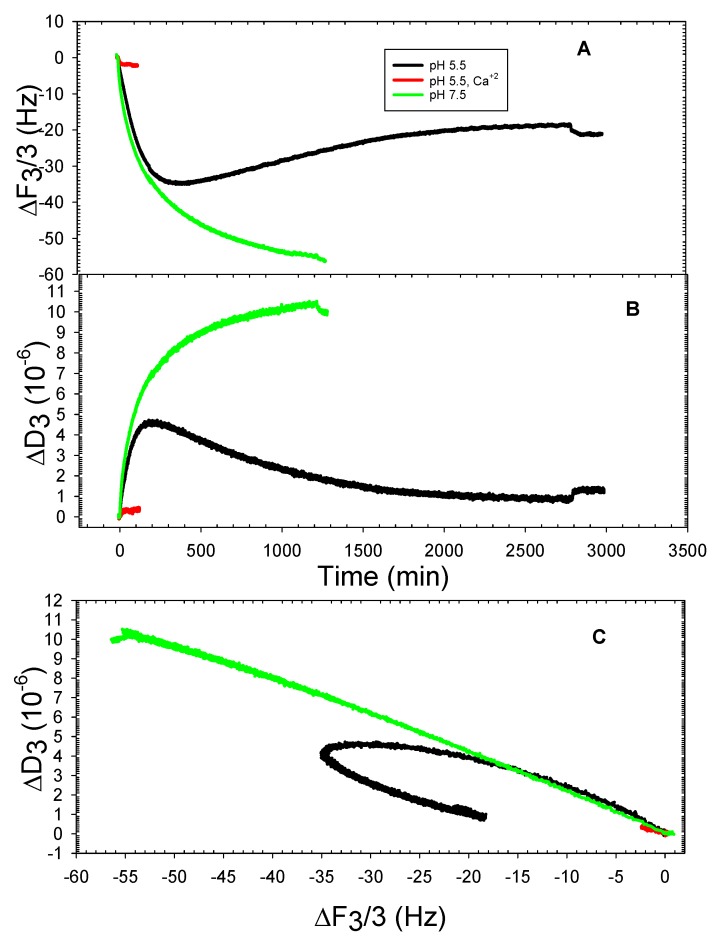
Representative QCMD frequency (**A**) and dissipation (**B**) shifts of DOPPE adsorption onto silica at pH 5.5 and pH 7.5 in the absence and presence of 2 mM CaCl_2_; ΔF vs. ΔD plot (**C**).

Figure 5 highlights the structural differences between DOPPE, DOPT, and DOPHT, where there was either zero, one, or two hydroxyl groups, respectively, attached to the phenyl moiety of the lipid headgroup. Liposomes containing 100% DOPPE exhibited the ability to adhere to silica at both pH 5.5 and pH 7.5, but not pH 10. The DOPPE liposomes required five hours to adhere at a maximum coverage before rupturing to form a supported bilayer at pH 5.5 in the absence of calcium ions. Rupturing of these liposomes to form a supported bilayer required more than an additional 24 h. The final frequency and dissipation shifts were approximately −22.5 Hz and 1.3 × 10^−6^ (Figure 5A,B). The final frequency values were well within the range typically found for thin, rigid supported bilayers [10]; however, the dissipation value was more indicative of a supported bilayer with excess water still trapped within or underneath it [18]. It is clear from the ΔF-ΔD plot that liposomes crowded the surface and formed a rigid liposomal layer, as indicated by the nearly constant dissipation shift values and increasing frequency shift values (Figure 5C, black line). The DOPPE liposomes under the buffer conditions of pH 7.5 in the absence of calcium ions required more than 16 h to adhere to the surface intact and without deforming or compacting together (Figure 5A,B, green line). The ΔF-ΔD plot indicated no deformation occurred during the adsorption (Figure 5C, green line). The DOPPE liposomes at pH 5.5 and in the presence of calcium ions marginally adhered intact in about 150 min (Figure 5, red line).

**Figure 6:**
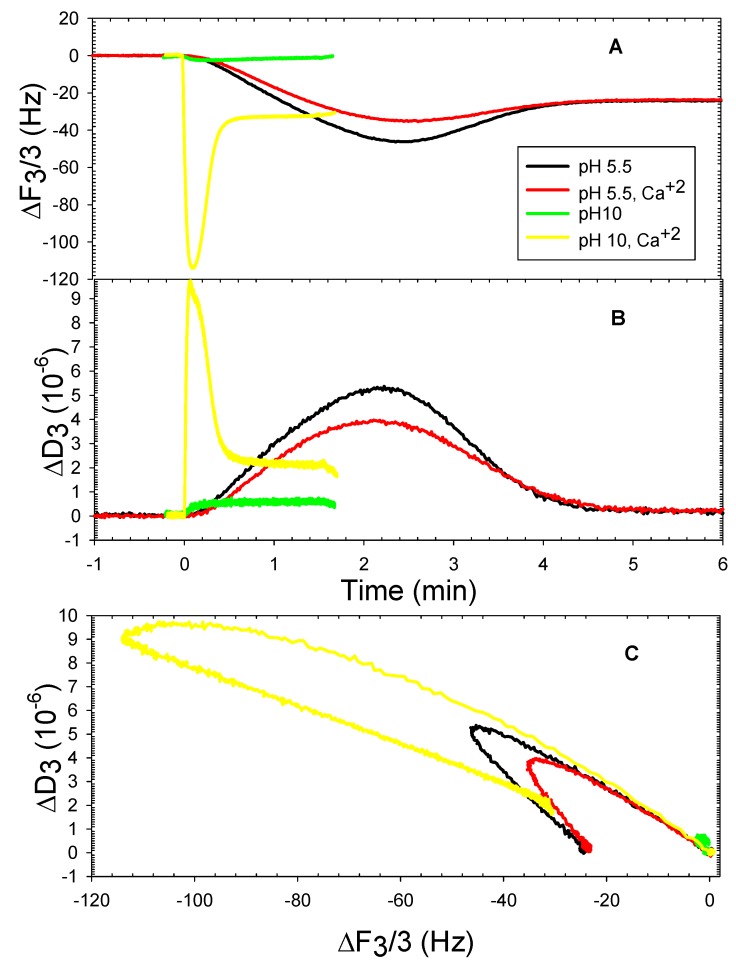
Representative QCMD frequency (**A**) and dissipation (**B**) shifts of DOPC adsorption onto silica at pH 5.5 and pH 10 in the absence and presence of 2 mM CaCl_2_; ΔF vs. ΔD plot (**C**).

Comparatively, the zwitterionic liposomes made of DOPC adhered to the silica surface and ruptured to form supported bilayers at pH 5.5 in the presence and absence of calcium ions, and at pH 10 in the presence of calcium ions (Figure 6). The exception was at pH 10 and in the absence of calcium ions, where DOPC liposomes only marginally adhered. It is noteworthy that DOPC liposomes formed a supported bilayer in less than a minute in the presence of calcium ions at pH 10, which was much faster than under the conditions previously reported for near neutral buffers [8,18,19]. The ΔF-ΔD plots (Figure 6C) show the typicalcusp shape as supported bilayers formed under the explored buffer conditions of pH 5.5. The cusp shape for conditions in the presence of calcium ions at pH 10 was a flattened cusp similar to that of DOPPE liposomes under the same conditions. This suggested that pH 10 may induce liposomal deformation prior to rupturing to form a supported bilayer. 

**Figure 7:**
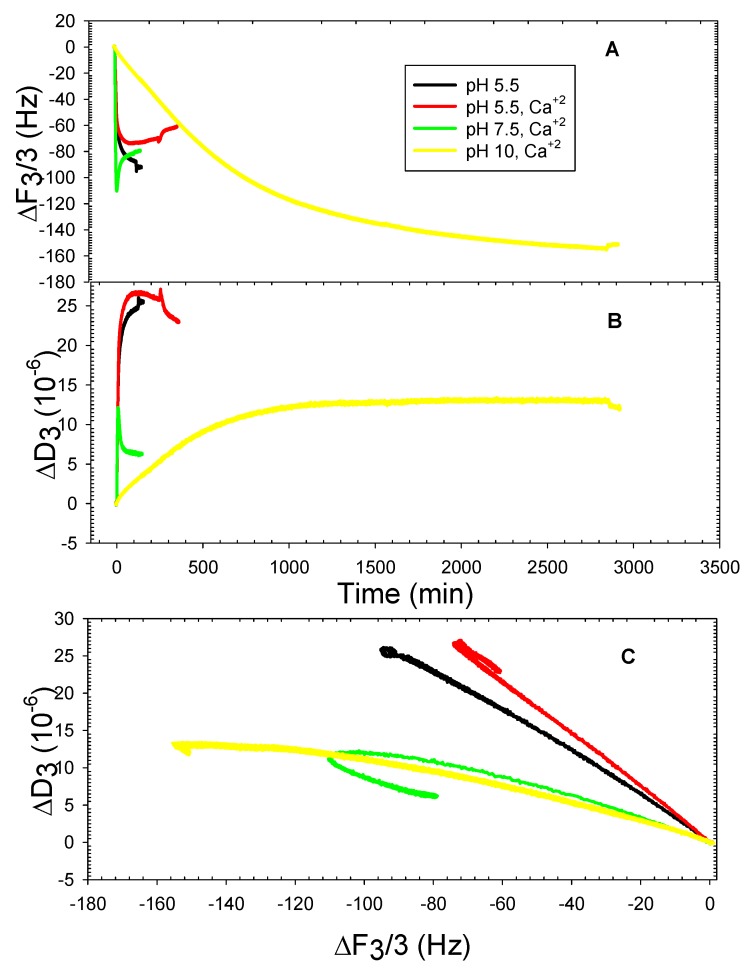
QCMD frequency (**A**) and dissipation (**B**) shifts representation of DOPG adsorption onto silica at pH 5.5, pH 7.5 and pH 10 in the absence and presence of 2 mM CaCl_2_; ΔF vs. ΔD plot (**C**).

Liposomes formed from the negatively-charged phospholipid DOPG formed supported bilayers only in the presence of calcium ions at both pH 5.5 and pH 7.5 (Figure 7A,B). Intact liposomes adsorbed to form a supported liposomal layer at pH 5.5 in the absence of calcium ions and at pH 10 in the presence of calcium ions. The difference in the two liposomal layers was that liposomes deformed at pH 10 (Figure 7C). The fact that neither DOPT or DOPHT liposomes adsorbed under pH 10 conditions is not too surprising, considering that each are expected to be fully deprotonated at pH 10, as stated above. Their full deprotonation would leave both of their phenolic rings at their highest negative charge. Taking this into consideration, and the expectation that the silica surface is also at its fullest negative charge, suggests that the repulsive nature between highly negatively-charged silica and DOPT or DOPHT liposomes is too great to overcome any shielding that salt ions may provide; therefore, no liposomes adsorb onto silica. This appears to be the case also for DOPPE liposomes. Calcium ions are, however, are able to shield DOPC, and DOPG enough for liposomes made of either are able to adsorb onto and form a supported bilayer or liposome layer, respectively, at pH 10.

## 4. Conclusions

Liposomes made from either 100 percent DOPHT, DOPT, or DOPPE were studied for liposomal and bilayer properties at pH 5.5 and pH 10. Dynamic light scattering and zeta potential measurements revealed that both size and surface charge were pH-dependent, with the greatest sense of stability occurring at pH 5.5 or pH 10. Quartz crystal microbalance with dissipation monitoring (QCMD) measurements revealed that only DOPPE liposomes would adsorb onto silica and form a supported bilayer at either pH studied, and both DOPHT and DOPT formed supported liposome layers. The fact that DOPHT and DOPT liposomes remained intact when contacting such a reactive surface known to induce supported bilayer formation strongly suggest that these liposomes would be extremely resistant to interaction in other solutions conditions, and are highly favored for adaption to the food industry.
